# Sparse and Random Sampling Techniques for High-Resolution, Full-Field, BSS-Based Structural Dynamics Identification from Video

**DOI:** 10.3390/s20123526

**Published:** 2020-06-22

**Authors:** Bridget Martinez, Andre Green, Moises Felipe Silva, Yongchao Yang, David Mascareñas

**Affiliations:** 1 Los Alamos National Laboratory, Los Alamos, NM 87544, USA; bmartinez26@ucmerced.edu (B.M.); andre_green@lanl.gov (A.G.); 2 Applied Electromagnetism Laboratory, Universidade Federal do Pará, R. Augusto Corrêa, Guamá 01, Belém, 66075-110 Pará, Brazil; moises.silva@icen.ufpa.br; 3 Department of Mechanical Engineering-Engineering Mechanics, Michigan Technological University, Houghton, MI 49931, USA; yyang14@mtu.edu

**Keywords:** compressive sensing, blind source separation, random projection, sparse reconstruction, phototoxicity, nonlinear filtering, cryptography, privacy-preserving structural health monitoring (SHM), 5G network

## Abstract

Video-based techniques for identification of structural dynamics have the advantage that they are very inexpensive to deploy compared to conventional accelerometer or strain gauge techniques. When structural dynamics from video is accomplished using full-field, high-resolution analysis techniques utilizing algorithms on the pixel time series such as principal components analysis and solutions to blind source separation the added benefit of high-resolution, full-field modal identification is achieved. An important property of video of vibrating structures is that it is particularly sparse. Typically video of vibrating structures has a dimensionality consisting of many thousands or even millions of pixels and hundreds to thousands of frames. However the motion of the vibrating structure can be described using only a few mode shapes and their associated time series. As a result, emerging techniques for sparse and random sampling such as compressive sensing should be applicable to performing modal identification on video. This work presents how full-field, high-resolution, structural dynamics identification frameworks can be coupled with compressive sampling. The techniques described in this work are demonstrated to be able to recover mode shapes from experimental video of vibrating structures when 70% to 90% of the frames from a video captured in the conventional manner are removed.

## 1. Introduction

Ensuring the reliable operation of critical infrastructure is going to require that we have persistent, autonomous sensing and analysis solutions. These analysis solutions must be capable of detecting, localizing and quantifying the structural integrity of large-scale infrastructure in harsh environments such as marine structures (e.g., wind turbines) over many length scales from the crack size up to the size of an entire wind farm. Mission-ready solutions for these problems do not currently exist. State-of-the art structural health monitoring solutions are expensive to install, do not have the spatial resolution to detect small, but potentially catastrophic, damage in large-scale structures [1,2], and require large amounts of training data that generally is not even possible to collect. To date, a wide variety of techniques have been developed for structural health monitoring. These techniques include ultrasonic/acoustic-based techniques [3,4,5,6,7,8,9], eddy-current-based techniques [10,11,12], and microwave-based techniques [13,14,15]. These technologies are promising for specific structural health monitoring applications, however, they are not readily suited to being scaled up for applications such as monitoring the structural health of an entire city. Structural-health monitoring schemes based on single point-of-measurement contact sensors are particularly ill-suited to monitoring of large-scale infrastructure because the geometry of single point contact sensors is approximately zero-dimensional, while the geometry of the infrastructure itself is large in 3 dimensions. The substantial difference in dimensionality between the sensors and the infrastructure to be monitored implies that a very large number of contact-based sensors will be required to fully observe the structure-of-interest, which is not practical for many applications because of the cost associated with the individual sensors. 

Photogrammetric experimental mechanics techniques that make use of digital imagers hold a lot of promise for structural health monitoring applications primarily because the measurements taken by imagers is inherently 2 dimensional. One of the main reasons being that it is much easier to get dense sensor coverage of a 3-dimensional structure using 2-dimensional sensing techniques as opposed to the 0-dimensional nature of contact sensors such as strain gauges or accelerometers. Furthermore, the recent emergence of 5G networking capabilities [16,17,18] and low-power, embedded graphical processing unit (GPU) computing resources [19] makes it feasible to think about deploying wireless networks of video-based sensor nodes across an entire city or other forms of large-scale infrastructure such as wind farms. In these video-based sensor nodes preprocessing of data can occur locally at the sensor node and then the data can be transferred to cloud computing resources or a central server to aggregate the data from all the sensor nodes. Video-based experimental mechanics measurements [20], combined with advanced image processing algorithms such as optical flow [21,22,23], motion magnification [24,25,26], and digital image correlation (DIC) [27,28], have been successfully demonstrated by the structural dynamics [29,30,31,32,33,34,35] and health monitoring (damage detection) communities [36]. This work hopes to provide sampling strategies that help facilitate the deployment of video-based experimental mechanics techniques to the field. 

Full-field, high-resolution, unsupervised learning-based structural dynamics identification frameworks based on decomposition algorithms such as principal components analysis and blind source separation have recently emerged as an alternative, non-contact optical measurement methods to achieve full-field, high-resolution structural dynamics measurements, which may significantly improve structural dynamics analysis and health monitoring. A recently proposed method [37], termed full-field video dynamics method, has been found efficient and can be implemented automatically. It exploits the physical relation between the unsupervised machine learning models (and the structural dynamics models to model and process the high-dimensional pixel measurements and motion information. Through a family of unsupervised machine learning algorithms (principal component analysis for dimension reduction and blind source separation for modal decomposition), it is able to perform automated, unsupervised, and efficient identification of the output-only structure’s modal frequencies, damping ratios, and full-field (full-pixel-resolution) mode shapes. These techniques can make use of digital video cameras that are relatively low-cost, agile, and provides simultaneous, very high spatial resolution measurements where every pixel effectively becomes a measurement point on the structure. These techniques show potential to be adequately sensitive and robust to environmental noise under well understood limitations. Mode shapes and natural frequencies have from a structure with vibrational amplitude of only 50 µm at a distance of 1.4 meters, while the imager was traveling at 39 mm/s [38,39]. These techniques can also be used to detect changes in structural stiffness representative of damage that is nearly an order of magnitude smaller than the nearest competitor using an array of contact sensors, resulting in significantly less costly measurements in comparison [40]. Furthermore, these loss-of-stiffness/damage detection techniques only rely on analyzing the curvature of the mode shapes identified from the video. There is no need to collect a calibrated displacement measurement from the video. In addition, full-field, high-resolution, unsupervised learning-based structural dynamics identification techniques have been utilized for inferring the load being applied to a structure [41]. If these techniques are made robust for forward deployment then it is possible to envision being able to use them to quantitatively characterize the structural integrity of critical infrastructure. 

It is of note that the video of vibrating structures is inherently very sparse. Typically video of vibrating structures has a dimensionality consisting of many thousands or even millions of pixels and hundreds to thousands of frames. However, the motion of the vibrating structure can be described using only a few mode shapes and their associated modal coordinates. This means that the video measurement potentially contain large amounts of redundant data that increase the computational demand for video dynamics processing.

In this study we explore emerging techniques for sparse and random sampling such as compressive sampling (CS) [42,43,44] to perform efficient full-field output-only modal identification on video in a non-uniform, low-rate (compressive) sampling framework. This framework is based on an intimate combination of compressive sampling theory with blind source separation (BSS)-based [45] techniques, implemented through a low-rate, random sampling of the frames. CS enables low-rate random sampling, and has been demonstrated in structural dynamics and health monitoring applications [46,47,48,49,50,51]. Compressive sampling is able to exactly recover a sparse signal from far fewer incoherent random measurements than is suggested by the Nyquist sampling theorem. Recovery of the original measurement from the compressed samples is achieved by solving a convex *ℓ*_1_-minimization optimization problem, which exploits the sparsity of the signal over an appropriate dictionary of signals (e.g., Fourier basis). The compressive sampling approach is applicable to the modal analysis problem, since the targeted monotone modal responses are spectrally most sparse over a Fourier representation, which is maximally incoherent with the non-uniform random sensing space.

This work presents how full-field, high-resolution, structural dynamics identification frameworks based on decomposition algorithms such as principal components analysis and blind source separation can be naturally and intimately coupled with compressive sampling techniques. The resulting low-data rate, random sensing framework can be summarized as follows. First, during the image/frame acquisition step, the frames are acquired using a conventional, uniform frame-rate imager. Next, frames are randomly selected for removal. The low-rate randomly sampled time series of motion for the pixels observing the motion of the vibrating structure are then directly decoupled by using principal component analysis and blind source separation algorithms, resulting in the recovery of a mode shape matrix, as well as the decoupled low-rate random samples of the modal coordinates. From the low-rate random samples of each modal coordinate, the L1 minimization recovers the original high-rate modal response, thereby enabling estimation of the frequency and damping ratio. Using this approach the mode shapes can be obtained directly without any change to the blind-source separation procedure for modal identification. However, if the modal coordinates are desired, an L1 minimization problem must be solved for each modal coordinate which requires knowledge of the measurement matrix. It is also possible to perform the L1 minimization step after using PCA and prior to performing blind source separation. The second approach tends to result in better performance, but it requires solving an L1 minimization problem for each PCA component retained to obtain both the mode shapes and modal coordinates. Solving these L1 minimization problems also requires knowledge of the measurement matrix. Both approaches will be demonstrated in this work. It must be noted that in this work, frames are captured from a conventional uniform frame rate imager and then frames are randomly removed. However other hardware or software process that results in frames randomly captured in time could also be analyzed using the techniques presented in this work.

The contributions of this work include the presentation of a technique for reconstruction of mode shapes and modal coordinates from randomly sampled frames using L1 minimization. Another contribution is the demonstration that algorithms that solve the blind source separation problem can directly recover high-resolution mode shape information from randomly-sampled frames of video of vibrating structures without conventional L1 minimization, without knowledge of the measurement matrix, and without temporal order. A mutual-information-based algorithm for detecting and removing corrupted frames from video that would otherwise disrupt modal identification is presented. Finally, a relationship between structural dynamics, compressive sampling and cryptography is presented to illuminate a path toward privacy-preserving structural health monitoring and process monitoring. This work demonstrates that by using compressive sampling it is possible to reduce the number of frames needed to perform blind-source separation-based structural dynamics identification by about an order of magnitude. As a result, the approach described in this work is very attractive for deployment on video-based wireless sensor nodes for structural health monitoring. 

## 2. Algorithm for Full-Field, High-Resolution Modal Identification from Video of Vibrating Structures

**Figure 1:**
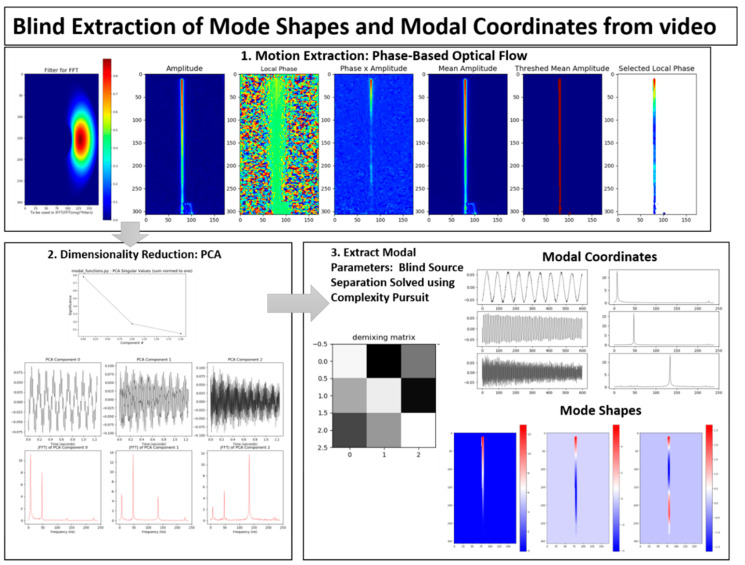
Overview of full-field principle components analysis (PCA)/blind source separation (BSS)-based blind source separation algorithm.

The output-only modal analysis algorithm for extracting full-field, high resolution structural dynamics information from video of vibrating structures can be summarized as follows [37], with Figure 1 providing a pictorial representation of these three steps using a video of a vertical cantilever beam as an illustrative example.

1. Convert the intensity measurements at each pixel into a quantity that maps to the displacement at each pixel. This transformation can potentially be accomplished in a variety of ways. For in-plane motion, phase-based optical flow [22,52,53], provides a useful means to perform the conversion from pixel intensity to displacement. Ultimately this procedure is applied across the time-span of the video and the result is a time series of displacement for every pixel. This step is illustrated in Figure 1 in the box labeled, “1. Motion Extraction: Phase-based Optical Flow.”
2. Perform principle components analysis (PCA) across all of the displacement time series at each pixel in order to reduce the dimensionality of the data. It is expected that the number of non-trivial eigenvalues calculated from the displacement time series for each pixel generated from a video of a vibrating structure will be of the same order as the number of mode shapes that are observable from the displacement time series. For this reason it is only necessary to retain the number of top eigenvectors that correspond roughly to the number of non-trivial singular values. It is important to note that the principle components that are calculated in this step correspond to time series of displacement captured by the pixels in the image. Because principle components are eigenvectors these displacements are scaled arbitrarily. There are no strict units of measure associated with the principle components at this step. This step is illustrated in Figure 1 in the box labeled, “2. Dimensionality Reduction: PCA.”
3. We now take advantage of the observation that the canonical form of the blind source separation problem has exactly the same form as the linear, multi-degree-of-freedom, structural dynamics equations when written in the modal form [54,55]. If the blind source separation problem is solved using the displacement time series extracted from the top principle components obtained from the principle components analysis in step 2, the columns of the mixing matrix provided by a blind source separation solution technique will yield the mode shapes which can be up-projected to the full-frame size at high-spatial resolution using the projection matrix obtained from the PCA step. The resulting individual signal sources correspond to the modal coordinates for each mode. It is important to note that because the modal coordinates are derived from the principle components in step 2, they do not have a strict unit of measure associated with them. The modal coordinates are simply arbitrarily-scaled displacement values. Furthermore, this is an output-only experimental modal analysis technique. There is no input measurement that can be used to scale the modal coordinates in a calibrated manner. Thus far complexity pursuit [56] has been found to be an attractive algorithm for solving the blind-source separation problem for structural dynamics applications. This step is illustrated in Figure 1 in the box labeled, “3. Extract Modal Parameters: Blind Source Separation Solved Using Complexity Pursuit.”

An additional, accessible summarization of these steps without the compressive sampling component can be found in [38]. After applying these steps to the video the result is high-resolution mode shapes and modal coordinates that can be used to estimate resonant frequencies and damping ratios. This approach to structural dynamics identification is attractive because it is conceptually easy to understand and the mode shapes provided have spatial resolution on the order of the pixel spot size. Furthermore, the algorithm lends itself to being automated which makes it attractive for long-term monitoring of structures. 

**Figure 2:**
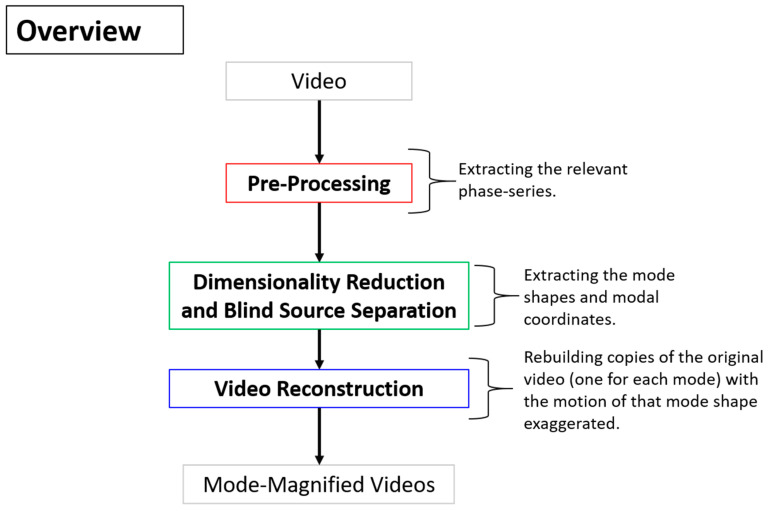
Color-coded overview of the main steps in the video dynamics algorithm.

**Figure 3:**
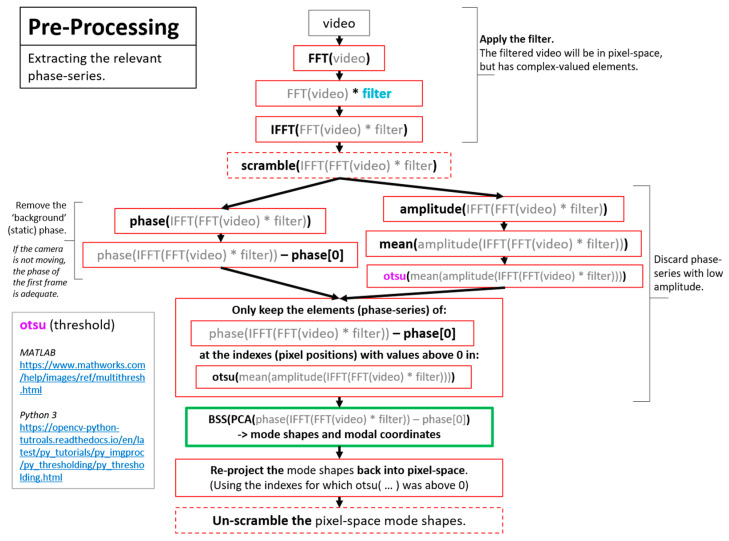
Pre-processing for motion extraction from pixel intensity values [22,52,53,58,59].

**Figure 4:**
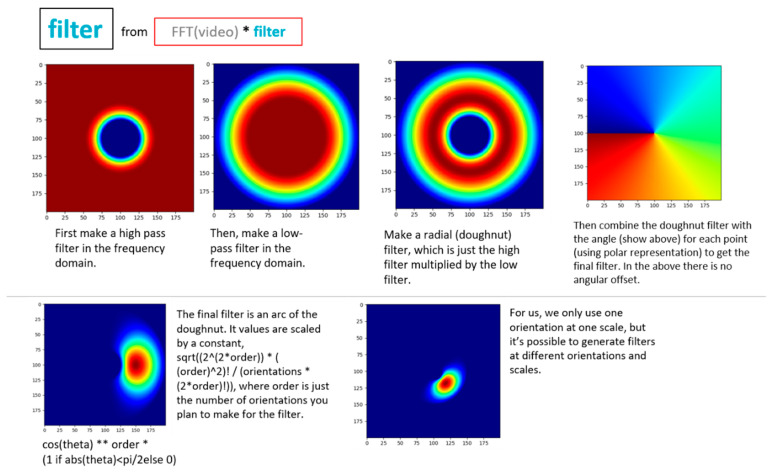
Overview of process for building filter to perform phase-based optical flow [22,52,53,57,58,59].

**Figure 5:**
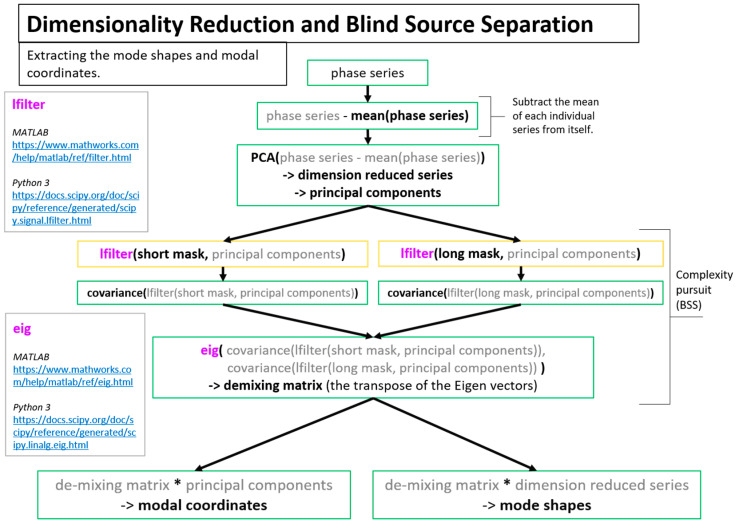
Dimensionality reduction and blind source separation to extract mode shapes and modal coordinates.

**Figure 6:**
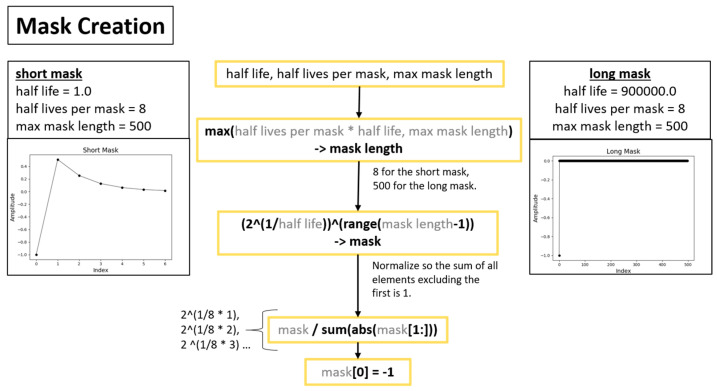
Overview of mask creation for implementing the complexity-pursuit solution to the blind source separation problem.

**Figure 7:**
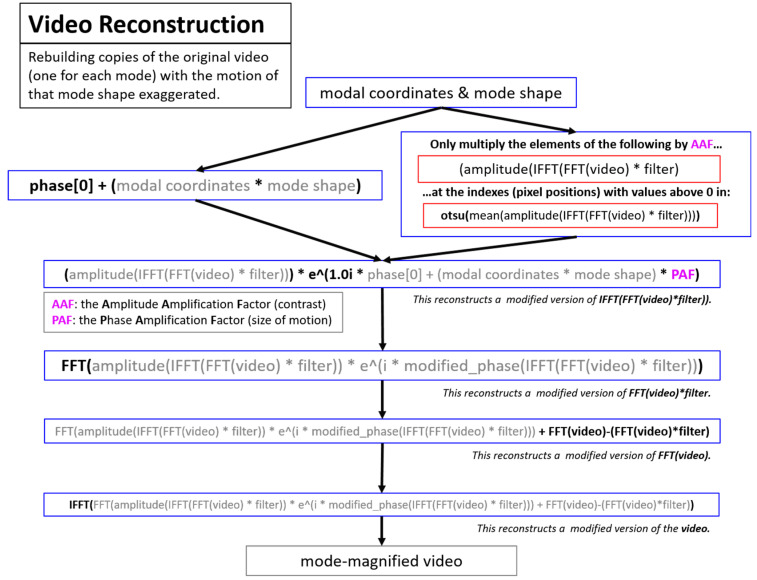
Overview of the process to perform motion magnification to visualize the mode shapes. This process is heavily inspired by the motion magnification process found in [52].

A more detailed overview of the steps in the algorithm for extracting full-field, high resolution structural dynamics information from video of vibrating structures without the compressive sampling component will now be provided. Figure 2 shows a color-coded overview of the entire algorithm and serves as a map for the remaining figures describing the algorithm. For the purpose of facilitating the detailed description of the algorithm, the algorithm has been split into 3 main components. The first component is called pre-processing (Figure 3). Additional details on the filters used for pre-processing are provided in Figure 4. The second component is referred to as “Dimensionality Reduction and Blind Source Separation” (Figure 5). Additional details on the masks used to perform the blind source separation are provided in Figure 6. The third component is referred to as “Video Reconstruction” (Figure 7). These components will now be elaborated upon.

The pre-processing component (Figure 3) is primarily concerned with extracting a time series that maps to displacement for every pixel in the image. This work makes use of phase-based optical flow [22], to extract a time series of phase values for every pixel in the image. In this case, phase maps to displacement in the direction determined by the filtering process. Intuitively phase is related to displacement in an image by the 2D Fourier shift theorem. Details on the filtering process used to implement phase-based optical flow and to specify the direction of the displacement of interest are shown in Figure 4. These images were created with the assistance of the utility files provided in [57]. Please note that Figure 3 also includes one box outlined in green that indicates where the dimensionality reduction and blind source separation fits in relation to the preprocessing step. The reason for this is that Section 10 will discuss techniques for performing structural dynamics in a privacy-preserving mode by scrambling the order the pixels in the image. 

The boxes with dashed lines with the labels “scramble,” and “unscramble,” indicate the points in the algorithm where scrambling of the order of the pixels would take place in the event it is desired to perform privacy-preserving structural identification. 

The dimensionality reduction and blind source separation process (Figure 5) is the portion of the algorithm that focuses on accomplishing the structural system identification. In this work dimensionality reduction is accomplished using principle component analysis and the structural system identification is accomplished by solving the blind source separation problem. For structural dynamics identification, complexity pursuit [56] has served as a good solution technique for the blind source separation problem [40,60,61]. Figure 6 provides details on the techniques used to create masks that are used in the complexity pursuit solution to blind source separation. 

Video reconstruction (Figure 7) is the process of magnifying the motion from individual modal components in order to visualize the mode shape motion in the context of the original video. This process is heavily inspired by the phase-based motion magnification work presented in [52]. The visualization process outlined in the video reconstruction component is not made use of in this work, but is included for completeness. 

## 3. Compressed Sampling Theory

Compressive sampling theory has had a large impact on the fields of applied math, signal processing, and statistics over the last decade. The reason for the large impact is that compressive sampling techniques allow for high-fidelity reconstructions of signals from fewer samples than the Nyquist sampling criterion would suggest. This means that less energy and bandwidth is required to capture, analyze and transmit data. It also means that memory requirements are reduced for storing data. In the context of the structural health monitoring problem, compressive sampling suggests approaches that could be used to reduce the energy consumption of sensor nodes deployed in the field to measure structural response. In turn this reduction in energy would lead to longer-lifetimes for the structural health monitoring sensor nodes. These advantages become even more clear if compressive sampling can be effectively applied to video-based sensor networks for structural health monitoring. This is because processing video typically requires significantly higher computational resources than processing conventional time series. This is the result of video having higher dimensionality than time series. 

Tutorials that provide a good overview of compressed sampling theory can be found in [44,62,63]. For those interested in performing a compressive sensing reconstruction on their own to gain an intuition for typical compressive sampling performance, a relevant example problem consisting of performing a reconstruction of a two-tone sinusoidal signal using compressive sampling can be found in [64] along with full Matlab code. In this example a 4096 point signal consisting of the sum of two sinusoids is reconstructed using only 128 measurements chosen randomly. This means the complete 4096 point signal can be accurately reconstructed using only 3.12% of the original measurements. This reduction in the required number of samples is very beneficial from the perspective of energy consumption associated with analysing, storing and transmitting these measurements.

(1) $$x=\sum_{i=1}^{N}s_{i}\psi_{i}\;\mathrm{or}\;\mathrm{in}\;\mathrm{matrix}\;\mathrm{form}\;\mathrm{as}\;x=\Psi s$$

Here Ψ is an orthonormal basis and “s” is the coefficients of the signal over the Ψ basis. For compressed sensing applications we only consider the case where *x* can be represented in a compressed form over some domain Ψ. Here a signal is considered compressible when the number of significant, non-zero elements in *s* is equal to *K*, and *K*<<*N*. *K* is referred to as the “sparsity” of the signal. A signal that is pure Gaussian noise is an example of a signal that would be excluded from consideration for compressed sampling. We now produce compressed sampling coefficients y by introducing a measurement matrix “Φ.”

(2) y=Φx=ΦΨs=Θs

The matrix Φ has M<<N rows. It now must be noted that (2) consists of an underdetermined system of linear equations. The major finding that underlies compressed sampling research is that assuming *K*<<*N* it is possible to reconstruct *x* from *y* assuming the measurements matrix possesses the restricted isometry property (RIP) and there exists a basis over which *x* is sparse [44,63]. Examples of matrices that satisfy the RIP property are matrices whose elements are drawn from a Gaussian distribution, and random subsets of the Dirac basis. In this work frames are randomly removed which corresponds to using random subsets of the Dirac basis. Structural vibration signals are often a good example of a sparse signal because in many cases they consist of a summation of a small number of sinusoidal components. The direct formulation of the problem of finding the vector *s* involves solving an optimization problem that tries to find the s with the minimum l_0_ norm. The challenge with the direct formulation is that l_0_ norm minimization is computationally expensive and it is numerically unstable [44]. However, it has been found that solving the l_0_-norm problem can be replaced with an l_1_ norm relaxation [62]. The l_1_ norm regularization problem is convex and thus efficient techniques exist to find solutions to this problem ([65]). The l_1_ norm regularization problem used to attempt recovery of the original signal *x* can be written as:

(3) $$\begin{array}{c} Minimize\\ s \end{array}\left({\left\|y-\Phi \Psi s\right\|}_{2}+\gamma {\left\|s\right\|}_{1}\right)$$

The *γ* parameter trades-off between the sparsity in the coefficients of *s* and the fidelity of the fit to the compressive samples *y.* When *γ* goes to 0 the problem simplifies to the conventional l_2_ norm problem. Prior work by the authors has shown that similar compressed sensing results are achieved using a range of *γ* values [66]. In this work *γ* is set to 0.01. Notably, *x* can be reconstructed from M measurements as governed by the relation [44]:

(4) $$M\geq cK*log\left(\frac{N}{K}\right)$$

Here *c* is a constant that has been found to be approximately equal to 4 based on empirical studies [44]. Equation (4) implies that a sparse signal *x* can be recovered using far fewer measurements than the number of elements in *x*. 

### 3.1. Recovery of Mode Shapes from Randomly Down-Sampled Measurements Using only Modal Identification

If one is particularly perceptive it is possible to see that the algorithm for full-field high-resolution structural identification inherently possesses properties of compressive sampling similar to those found in [47]. In fact, the algorithm in its current form allows for the automatic estimation of the mode shapes when only a randomly selected subset of the frames are used for analysis. Amazingly, it is not even necessary to know what the random measurement matrix is to achieve this benefit. The reason for this property is that the mode shape information is contained in the mixing matrix that is obtained when solving the blind source separation problem. The mixing relationship is theoretically not affected by the removal of frames. Therefore the theory suggests that frames can simply be removed and the mode shapes will still be faithfully recovered with no change to the algorithm and no need for any information of the mixing matrix. In contrast traditional applications of compressive sampling to structural dynamics data requires the measurement matrix be known in order to perform an L1 minimization step to recover the original signal from the compressively sampled signal [49]. To further clarify, the reason that mode shapes can be recovered automatically from randomly measured samples is related to the phenomena that allows this algorithm to operate even when the video is captured at a framerate below the Nyquist frequency of the structural vibrations [67]. To clarify, the canonical blind source separation problem can be written as:

(5) x(t)=As(t)

where *x*(*t*) is a column vector of the time series for each measurement available for analysis, *s*(*t*) is a column vector of time series associated with the sources generating the measured data, and *A* is the mixing matrix that indicates how the source time series are combined to form the measured time series. For comparison, the linear, multi-degree-of-freedom structural dynamics equations are written as:

(6) x(t)=Φq(t)

where *x*(*t*) is once again a column vector of the time series for each measurement available for analysis. In this case presumably displacement measurements are from a vibrating structure. *Φ* is a matrix whose columns are the structural mode shapes, and q(t) is a column vector of the modal coordinates. An examination of Equations (5) and (6) reveals that the mixing matrix *A*, and by extension the mode shape matrix *Φ* do not depend on the temporal nature of the measurements or the temporal nature of the generating sources/modal coordinates. As a result, if all that is needed is the high-resolution mode shapes, the blind source separation step will theoretically extract the mode shapes regardless of whether all the frames are present, or only a subset of the frames are present. The case of only a subset of the frames being present would correspond to the case where the compressive sampling measurement matrix is a random subset of the Dirac basis. In either case, the mixing relationship between the source signals is preserved and therefore the algorithm will theoretically return the correct mode shapes. Furthermore, unlike in conventional compressive sampling procedures there is no need to perform any kind of L1 minimization-based reconstruction step, there is no need to know the form of the mixing matrix, and there is no need to know the basis on which the signal is sparse. Solving the blind source separation problem automatically estimates the mode shapes without any of this information or extra steps. 

### 3.2. Application that Motivate Compressive Sampling for Video-Based Structural Dynamics

A primary motivation for the use of compressive sampling in video-based structural dynamics is that it offers the ability to deal with frames that have been corrupted, by removing their impact on the analysis. This is not a concern in laboratory environments, but it is a significant concern when considering the deployment of video-based wireless sensor networks for structural health monitoring. In the case of a field-deployed network of video cameras for structural health monitoring, it is possible that some frames will be corrupted by noise, or occluders such as birds flying across the frame. In some cases it is possible that frames will simply be dropped as a result of wireless communication link failures. Compressive sampling and sparse recovery techniques described in this work offer a solution to mitigate both dropped and corrupted frames.

Finally, applications such as live-cell imagery under a microscope are subject to photo toxicity and photo-bleaching effects [68]. In these applications repeated and continued exposure of live cells to light can damage and harm cells. Damage to cells is problematic when trying to answer scientific questions. Compressive sampling offers the possibility of reducing the duty cycle associated with the illumination used on the cells while video is taken to measure their structural dynamics. Compressive sampling for structural dynamics in as described in this work opens up the possibility of only capturing a small percentage of the frames in comparison to what the Nyquist sampling theorem suggests would be needed. Therefore the illumination on the cells could only be turned on during the capture of the small number of frames that would be needed. As a result, the light dose to the cell would be greatly reduced and the resultant light-induced damage to the cell would be minimized and the cells would remain healthy.

Applying compressive sampling techniques to video for high-resolution, full-field structural dynamics applications comes with many advantages associated with data compression, de-noising, and illumination requirements.

## 4. Analysis of the Sparse Nature of Video of Vibrating Structures

It has long been known that natural images make up only a very small subset of all possible images [64,69,70,71,72]. The subset of natural images tends to exhibit a lot of structure and as a result techniques such as data compression can feasibly be developed for this class of data. Video of vibrating structures is an even smaller subset of natural images. Linear structural dynamics theory says that structural vibrations can be described by a sum of mode shapes modulated in time by modal coordinate time series. It follows that a lower bound on the compression of a video of a structure undergoing linear vibrations is on the order of one frame per mode shape and one time series per mode shape. And in many cases it is probably possible to perform additional compression on the mode shape frames and modal coordinate time series.

**Figure 8:**
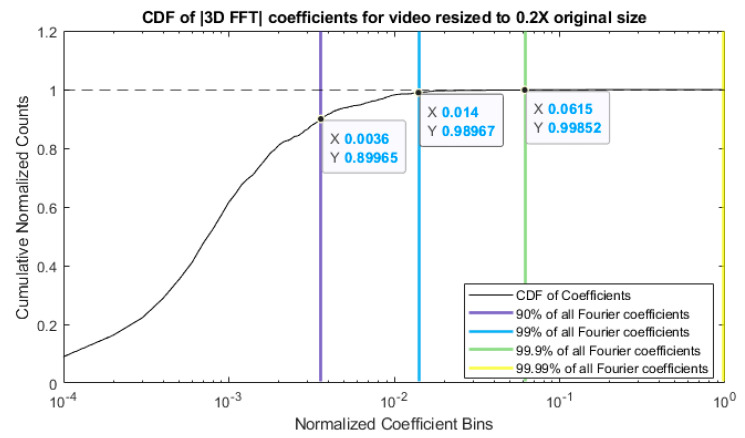
Cumulative density function of the normalized 3D Fourier coefficients of the vibrating cantilever beam video.

The sparsity of a video of a vibrating structure is now illustrated using an experimentally collected video of an aluminum cantilever beam subject to an impulse excitation at its base. It is known from prior analysis that 3 modes can be observed in this video [37]. Each video frame in the video is converted to grayscale and resized to 20% of its original size to match the size used in the analysis throughout this work. The dimensions of the original video are 216 × 384 pixels with a total of 600 frames captured at 480 fps, for a total of 49,766,400 values. After the resizing, the dimensions are 44 × 77 pixels with 600 frames (2,032,800 values). The 3D discrete Fourier transform is then taken of the resized video. The magnitude of the resulting complex coefficients are calculated and all the coefficients are normalized by the maximum value of the magnitude of the 3D Fourier coefficients. Next the histogram for the resulting coefficients sorted into 10,000 bins is found. The histogram values are normalized so their sum is equal to 1 and the cumulative sum across the bins is calculated. The cumulative density function of the magnitudes of the normalized coefficient values is then plotted as shown in Figure 8. When plotting the CDF figure, the cumulative sum is used, and the horizontal axis (upon which the normalized coefficient bins lie) is set on a log scale for visual clarity. The bin index of the cumulative sum values of 0.9, 0.99, 0.999, and 0.9999 are acquired and vertical lines are drawn at each of these indices. Each vertical line marks the fraction of the range of the Fourier coefficients below which some fraction of all of the absolute value Fourier coefficients lie. For example, the purple line indicates that ~90% of the coefficients lie in the bottom 0.3% of the total range of the Fourier coefficient’s absolute values. Likewise, ~99% of the Fourier coefficients reside in the bottom 1.4% of the total range for the Fourier coefficient’s absolute values, and so on for the green and yellow lines. This analysis reveals that video of a vibrating structure has a very sparse representation over a standard Fourier basis. It follows that analysis and data storage of video of vibrating structures should significantly benefit from sparse sampling methods such as compressive sampling and sparse reconstruction techniques.

## 5. The Impact of Corrupted Frames on Modal Identification Performance

**Figure 9:**
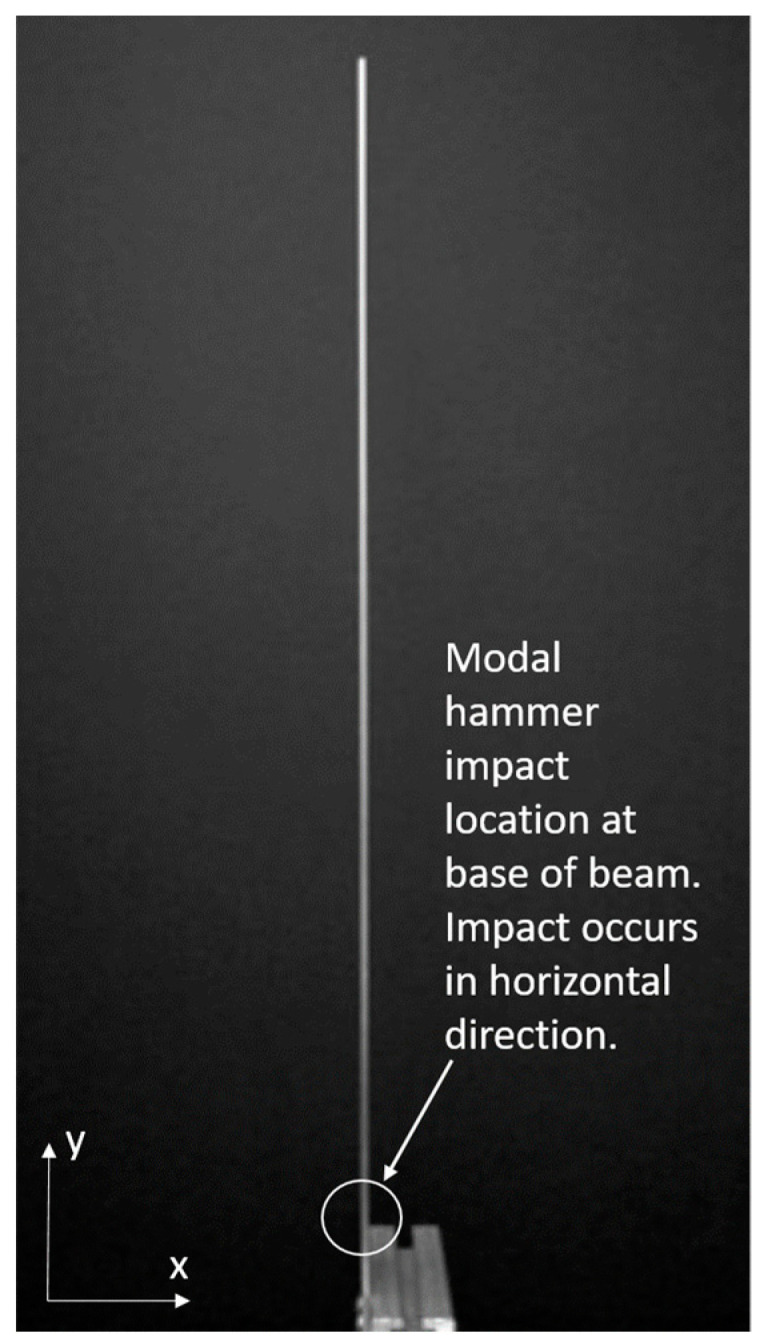
Cantilever beam experimental setup.

**Figure 10:**
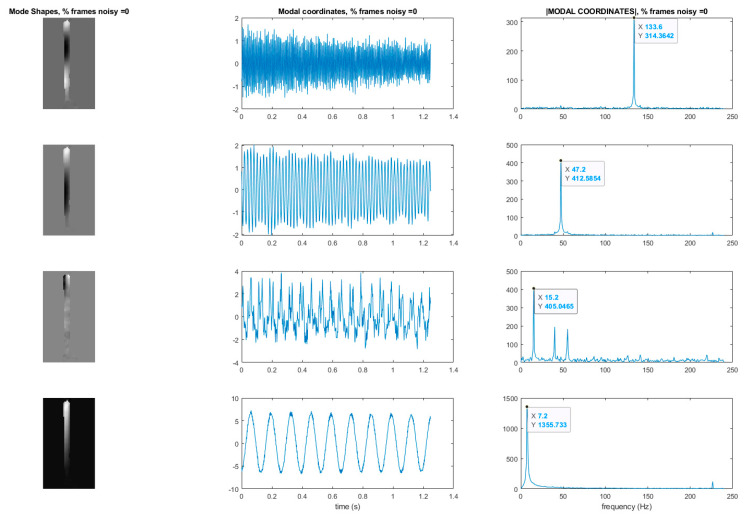
Mode shapes and modal coordinates of the cantilever beam recovered from the original video. *Y*-axis of the modal coordinate and the magnitude of the fft(modal coordinate) plots are arbitrarily scaled displacement.

This section may be divided by subheadings. It should provide a concise and precise description of the experimental results, their interpretation as well as the experimental conclusions that can be drawn. Now consider the results when the full-field, high-resolution, modal identification algorithm is applied to an experimentally-captured video of a cantilever beam that has been excited with a hammer impact at the base (Figure 9). The video is 216 × 384 pixels with a total of 600 frames captured at captured at 480 frames per second. It is known from prior analysis that the cantilever bean exhibits 3 modes. The natural frequencies of these modes have been measured to be: 7.5 Hz, 48.2 Hz, and 133.6 Hz [37]. The phase-based optical flow was only carried out in the horizontal (x) direction because the vibration motion was almost exclusively in the horizontal direction. The PCA and CP algorithm were directed to find 4 modes. It is expected that one of the modes output by CP will be a computational mode that is simply an artifact of the analysis. The resulting mode shapes, modal coordinates, and magnitude of the fft(modal coordinates) can be found in Figure 10. Please note that the modal coordinates obtained in this analysis are derived from principle components of the displacement time series associated with each pixel. Because principle components are eigenvectors of a covariance matrix they do not have an absolute scale. Therefore there are no strict units of measurement associated with the modal coordinates reported in this work. The captions under the figures indicate the vertical axis of the modal coordinates in both the time and frequency domain are simply arbitrarily-scaled displacement values. An examination of the mode shapes reveals that they have the same structure as the analytical solution for the mode shapes of a cantilever beam. A strong, purely sinusoid modal coordinate is associated with each mode shape. The pure tone nature of these modal coordinates provides additional evidence that these are in fact mode shapes. The 2nd from the bottom component shown in Figure 10 is not a single pure tone like the others. Furthermore, the mode shape shown 2nd from the bottom does not have 3 prominent inflection points as would be expected in a 3rd mode of a cantilever beam. For these reasons we assume that the comment associated with the data displayed 2nd from the bottom in Figure 10 is a computational mode that can be ignored. Based on a frequency domain analysis of the modal coordinates obtained from analysis of the video, the first three natural frequencies of the beam are 7.2 Hz, 47.2 Hz, and 133.6 Hz. Note that each of the 3 modal coordinates only have energy at a single tone which provides good evidence these are structural modes. 

**Figure 11:**
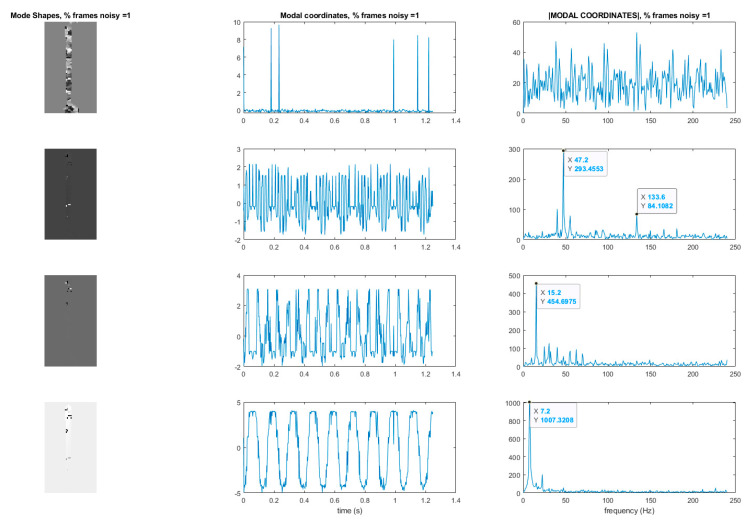
Mode shapes and modal coordinates of the cantilever beam recovered when 1% of the frames are randomly replaced by static frames. *Y*-axis of the modal coordinate and the magnitude of the fft(modal coordinate) plots are arbitrarily scaled displacement.

**Figure 12:**
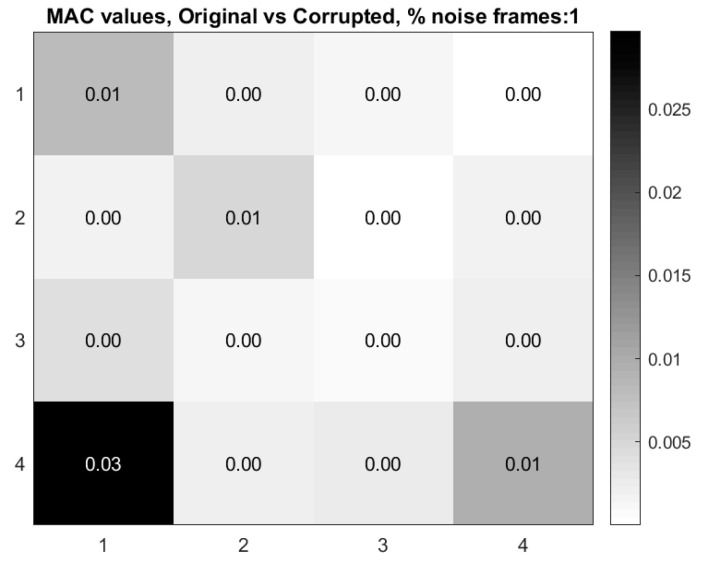
Modal assurance criterion (MAC) values comparing modes recovered from the original video with video for which 1% of the frames from the original video are replaced with Gaussian noise frames.

Now consider the case where 1% of the 600 frames in the video of the vibrating cantilever beam are replaced by frames consisting of only Gaussian-distributed noise. Figure 11 shows the mode shapes, modal coordinates, and the magnitude of the fft(modal coordinates) recovered using the blind-source separation-based algorithm previously described. Figure 12 shows the modal assurance criterion (MAC) values comparing the mode shapes recovered from the original video and the video with 1% of the frames replaced by corrupted frames. An examination of these two figures quickly reveals that the modal identification algorithm has completely lost the ability to characterize the mode shapes and modal coordinates of the cantilever beam. Furthermore, only the natural frequency of the first mode is accurately captured. The loss in performance of the algorithm is presumably on account of the random introduction of the Gaussian noise frames. This example demonstrates the importance of detecting and removing frames that corrupt the video being used for structural dynamics characterization. Corruption of frames can be the result of noise or electromagnetic interference of the imager, dropped frames, or occluders such as birds or vehicles passing through the frame during the measurement. The next section will discuss a mutual-information-based approach for detecting frames which are corrupted. 

## 6. Mutual Information-Based Technique for Detection of Corrupted Frames

Based on the results in Section 6, it is now clear that including corrupted frames in the measurements input to the high-resolution, full-field modal identification algorithm for video has the potential to greatly reduce the quality of the modal identification. It is clear there is a need to create an algorithm for detecting the presence of corrupted frames. An algorithm based on mutual information is now presented for detecting the presence of corrupted frames in the video. 

Mutual information is used to decide whether a given frame is corrupted by evaluating whether the sum of the normalized mutual information between said frame and all others lies below a particular threshold. The intuition behind this technique is that the average mutual information of a corrupt frame with all other frames will be low. More precisely, the contents of a corrupt frame do not significantly reduce the uncertainty in predicting the other frames of the video. This assumption is particularly valid when the corruption is caused by noise. 

Mutual information is defined as follows.

(7) $$I\left(X,Y\right)=\underset{x\in X}{\sum }\underset{y\in Y}{\sum }p_{X,Y}\left(x,y\right)\cdot \log_{2}\left(\frac{p_{X,Y}\left(x,y\right)}{p_{X}\left(x\right)\cdot p_{Y}\left(y\right)}\right)$$

The term *p_X_*(*x*) is the probability of observing *x* in *X*. In this application, it is the number of times pixels with values of *x* appear in the image *X*, divided by the total number of pixels. The term *p_Y_*(*y*) is the probability of observing *y* in *Y*. In this application, it is the number of times pixels with values of *y* appear in the image *Y*, divided by the total number of pixels. Finally, the term *p_X,Y_*(*x*,*y*) is the probability of observing *x* in *X* and *y* in *Y* simultaneously. In this application, it is the number of times *X* at pixel position (*a*,*b*) equals *x*, and *Y* at pixel position (*a*,*b*) equals *y* over all positions (*a*,*b*), divided by the total number of pixel positions.

### 6.1. The Problem of Non-Normalized Mutual Information

It is insufficient, however, to examine the raw value returned by mutual information to detect aberrant frames. This is because mutual information varies both because of the relationship between the two signals and the signals themselves. For example, two similar signals may have little mutual information simply because one or both of the signals have little information. Likewise, two dissimilar, noisy signals may have high mutual information on account of both having a lot of information. Hence it is possible that a valid signal with little information may have less mutual information with another valid signal bearing little information than it does with a dissimilar or noisy signal bearing lots of information. 

To address this issue we normalize by the maximum of the information (formalized as Shannon Entropy) contained in either image. This normalization addresses both the issue of valid signals with low information having low mutual information, and noisy signals (with inherently high information content) having high mutual information. 

### 6.2. Proof that Division by Maximum Entropy Bounds Mutual information to [0, 1]

Mutual information is already non-negative—given the following identity:

(8) $$\ln\left(x\right)\geq 1-\frac{1}{z}\;for\;x>0$$

The knowledge that log_2_(*x*) is a constant multiple of ln(*x*), and hence may be swapped and back in painlessly:

(9) $$\log_{2}x=\frac{\ln x}{\ln2}$$

One may show the non-negativity of mutual information as follows:

(10) $$\begin{array}{l} I\left(X,Y\right)=\underset{x\in X}{\sum }\underset{y\in Y}{\sum }p\left(X,Y\right)\left(x,y\right)\cdot \ln\left(\frac{p\left(X,Y\right)\left(x,y\right)}{pX\left(x\right)\cdot pY\left(y\right)}\right)\\ \;\geq \underset{x\in X}{\sum }\underset{y\in Y}{\sum }p\left(X,Y\right)\left(x,y\right)\cdot \left(1-\frac{p\left(X,Y\right)\left(x,y\right)}{pX\left(x\right)\cdot pY\left(y\right)}\right)\\ \;=\underset{x\in X}{\sum }\underset{y\in Y}{\sum }p\left(X,Y\right)\left(x,y\right)-\underset{x\in X}{\sum }\underset{y\in Y}{\sum }pX\left(x\right)\cdot pY\left(y\right)\geq 0 \end{array}$$

To fix the upper bound on mutual information at 1, we divide by the entropy of *X* or the entropy of *Y*, whichever is the larger of the two. To demonstrate that the maximum value for mutual information is the maximum of the entropy of its constituent signals, consider that mutual information may be equivalently defined as:

(11) I(X,Y)=S(X)−S(X|Y)

where *S*(*X*) is Shannon entropy:

(12) $$S\left(X\right)=-\underset{x\epsilon X}{\sum }p\left(x\right)\cdot log2\left(p\left(x\right)\right)$$

*S*(*X*|*Y*) is the conditional Shannon entropy:

(13) $$S\left(X|Y\right)=\underset{y\in Y}{\sum }pY\left(y\right)\cdot \left[-\sum^{\text{​}}pX|Y(x|y)\cdot log2(pX|Y(x|y)\right]$$

To maximize the mutual information, it is necessary to select *Y* such that *S*(*X*|*Y*) is minimized.

It is evident that *S*(*X*|*Y*) is nonnegative:

(14) $$p_{X|Y}\left(x,y\right)\in \left[0,\;1\right]$$

Which implies that, regardless of the values of *x* and *y*,

(15) $$-p_{X|Y}\left(x,y\right)\cdot \log_{2}p_{X|Y}\left(x,y\right)\in \left[0,\;\infty \right)$$

As such, entropy is maximized for *X* when *Y* is chosen such that *S*(*X*|*Y*) is minimized.

This can occur when *Y* = *X*:

(16) $$S\left(X|X\right)=\underset{x\in X}{\sum }pX\left(x\right)\cdot \left[-\sum^{\text{​}}pX|X(x|x)\cdot log2(pX|X(x|x)\right]=0$$

Hence, *S*(*X*) is the maximum value for mutual information under this normalization scheme.

(17) I(X,Y)=S(X)−S(X|X)=S(X)

Insofar as mutual information is commutative (shown by observing all its operations to be commutative), the same argument applies by symmetry to *Y*.

In order to test the efficacy of the mutual-information-based corrupt frame detection algorithm, videos of the vibrating cantilever beam were generated in such a way that randomly selected frames were corrupted with noise. The different types of noisy videos were created by randomly selecting a percentage of the input frames and then inserting four different types of noise in each of the randomly selected frames. The noise was added individually, not simultaneously, so a separate noisy video was created for each noise type. Gaussian white noise, which commonly occurs during data acquisition, was simulated by drawing samples from a Gaussian distribution with zero-mean and variance of 0.01. The samples were then added over all the pixels in the selected frames. Speckle noise was simulated using samples drawn from a uniform distribution with zero-mean and a variance of 0.05. The samples were then added to all the pixels in the randomly selected frames. Note that these two types of noise correspond to additive noise cases. Also, for the purpose of adding the noise the pixel values, originally integers ranging from 0 to 255, were converted to double precision numbers and scaled from [0, 1] before adding the noise. After the addition of the noise samples, the noisy images were rescaled back to the original RGB scale of 0–255 and converted back into integers. 

Impulsive salt and pepper noise with a density of 0.05 was also considered. The density of 0.05 indicates that 5% of all the pixels in the randomly selected frames are replaced by either 0 or 255. Generally this noise occurs during the analog-to-digital conversion process, as a result of damage to the pixels. Finally, video frames corrupted by Poisson noise, also known as photon shot noise, were generated. Poisson noise is distinguished from the previous types of noise, in the sense that the noise is produced such that it is proportional to the image intensity. Poisson noise was also applied to the randomly selected frames.

**Figure 13:**
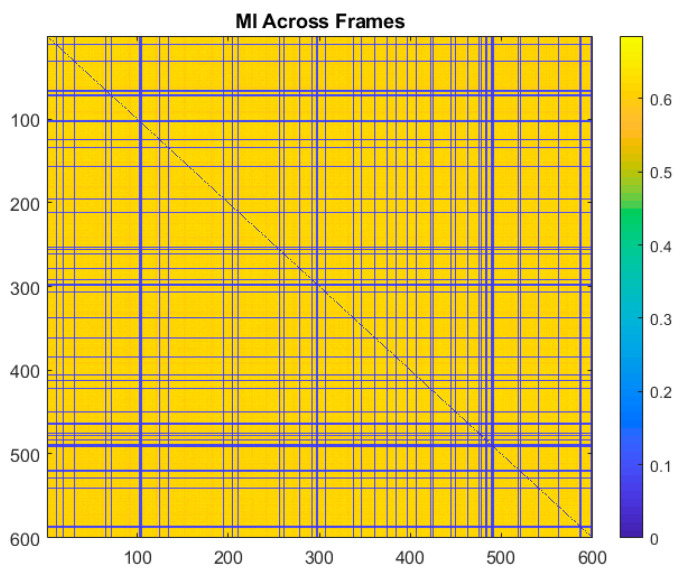
Normalized mutual information calculated between all the frames and one another.

**Figure 14:**
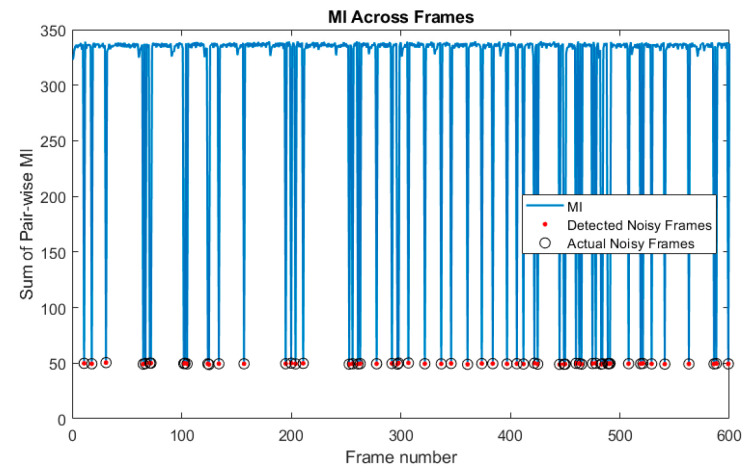
Sum of normalized mutual information calculated between all the frames and one another. This information is used to detect the presence of corrupted frames so they can be removed.

Videos of the cantilever beam corrupted by the four types of noise previously described were generated. The videos were generated such that 1.0%, 10%, 25%, 50%, 70%, 90%, 95%, and 99% of the frames were randomly selected and corrupted by noise. Throughout this paper results are presented for multiple values of frames being randomly removed in order to provide information on the robustness of the technique and to demonstrate where the performance drops off. The normalized mutual information was then calculated by the frames and the sum of the normalized mutual information was taken across all the frames. An example of a plot of the normalized mutual information for the video with 10% of the frames in the video corrupted by Gaussian noise is shown in Figure 13. The corresponding plot of the sum of the mutual information across all the frames is shown in Figure 14. In the normalized mutual information plot the dark blue lines indicate locations where mutual information between frames is low, which indicates the presence of a frame corrupted by noise. By using a simple threshold on the sum of the normalized mutual information, it is shown that it is possible to detect all of the frames corrupted by noise with no false positives or false negatives. In fact, the sum of normalized mutual information algorithm achieved 100% corrupt frame detection with no false negatives or false positives over all the noise types and percentages of frames corrupted considered. It is important to mention that the noise types considered in this analysis are significantly less severe in terms of signal-to-noise ratio across a frame than the case of randomly replacing frames with 100% Gaussian noise which was shown in Section 5 to completely damage the ability to perform modal identification. It follows then that the normalized mutual information detector is expected to be able to detect frames that are corrupted enough to completely degrade the performance of the modal identification. 

## 7. Use of L1 Regularization to Perform Modal Identification from Sparse Samples of Frames

**Figure 15:**
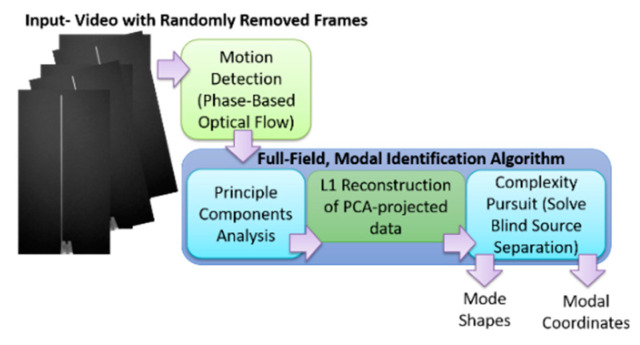
Algorithm for performing modal identification on video with frames randomly removed using L1 regularization.

**Figure 16:**
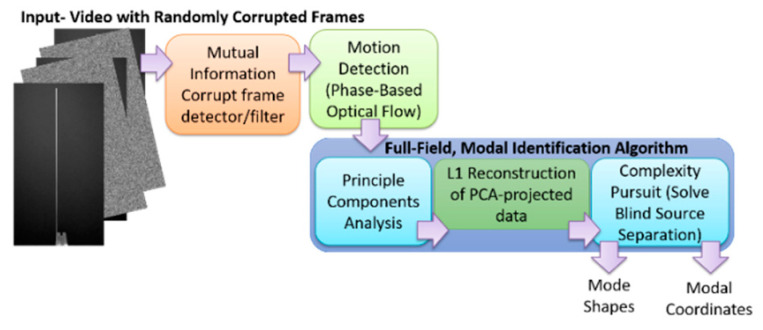
Algorithm for performing modal identification on video with randomly corrupted frames using L1 regularization.

**Figure 17:**
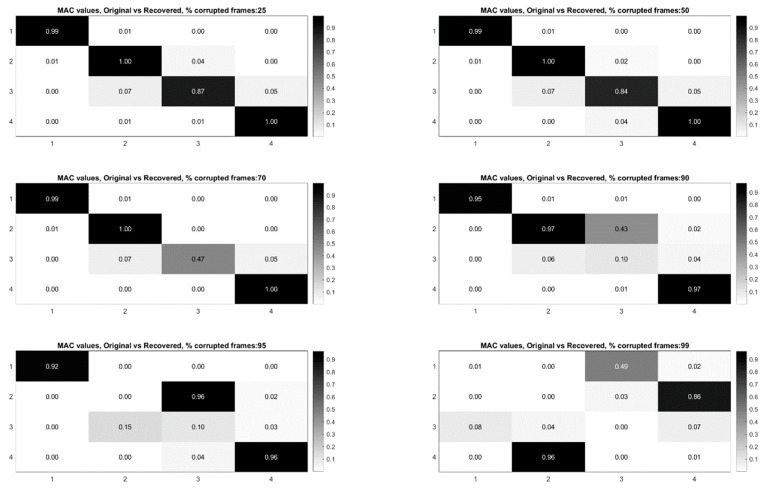
Modal assurance criterion values for the mode shapes recovered using L1 minimization on the PCA components. Each MAC matrix corresponds to a different percentage of corrupt/removed frames.

**Figure 18:**
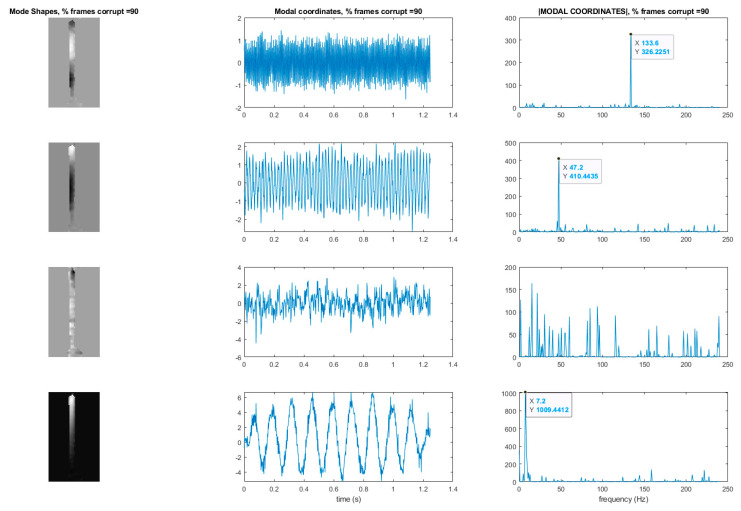
Mode shapes and modal coordinates for the case of L1 reconstruction after the PCA step. Results are shown for the case of 90% of the frames randomly removed. Y-axis of the modal coordinate and the magnitude of the fft(modal coordinate) plots are arbitrarily scaled displacement.

**Figure 19:**
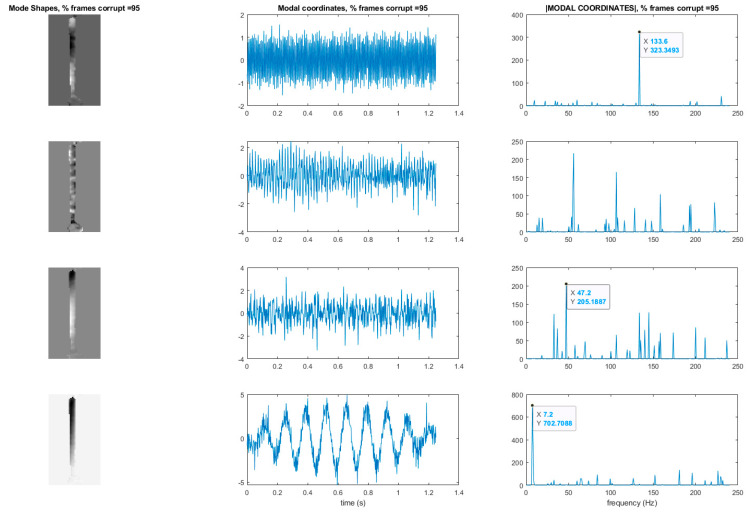
Mode shapes and modal coordinates for the case of L1 reconstruction after the PCA step. Results are shown for the case of 95% of the frames randomly removed. *Y*-axis of the modal coordinate and the magnitude of the fft(modal coordinate) plots are arbitrarily scaled displacement.

Now that a technique has been developed for detecting the presence of randomly corrupted frames in the video of vibrating structure, we now show techniques that allow these frames to be removed from the video and still perform full-field modal identification. These techniques also work for the case where frames are simply randomly removed from the video of the vibrating structure. In this section, the techniques rely on the use of L1 minimization algorithms as is typically found in compressive sampling problems. As a result the measurement matrix indicating how frames have been removed must be known. Figure 15 and Figure 16 illustrate the procedure for performing full-field, high-resolution modal identification from randomly sampled video using L1 minimization for the case of randomly sampled frames, as well as for the case of video frames being randomly corrupted respectively. The procedure for the two cases is identical except that in the case of the randomly corrupted frames the video is first run through the mutual information-based corrupt frame detection/filtering algorithm. In both cases, the remaining frames are then sent to the motion detection algorithm and the resulting motion time series at each pixel are then subjected to principle components analysis. In this work we only consider pixel time series associated with pixels that display motion. Considering only pixel that display motion reduces the amount of data that must be processed. A list of the “active” pixel locations is maintained and used to reconstruct the total mode shape in pixel coordinates at the end of the analysis. Next, the down projected, randomly sampled data is reconstructed using L1 minimization to bring the dimensionality of the pixel time series back up to equal the total number of frames in the original video. In this work the reconstruction is accomplished using a Fourier basis. The reconstruction is carried out for the total number of components the user chooses to search for. In this work, four components are always used. The four principle component analysis components are then fed into a complexity pursuit algorithm to solve the blind source separation problem. The output of complexity pursuit is the modal coordinates and the columns of the resulting mixing matrix contains the resulting mode shape information. The mode shapes are then up-projected to match the full pixel-space of the video. The list of pixel locations associated with the “active” pixels is used to sort the mode shape components into their appropriate locations in the pixel space. All remaining “inactive” pixel locations are set to 0. The modal assurance criterion values for differing percentages of corrupt/removed frames as compared with the mode shapes recovered from the original video are shown in Figure 17 [73]. First, it is worth noting that the MAC values remain about 0.9 for all three modes even when 90% of the 600 total frames are randomly removed. Even in the case where 99% of the frames are randomly removed, the MAC values stay above 0.9 for the case of the first two modes. In this case, the 3rd mode is much harder to discern. Figure 18 shows the mode shapes, modal coordinates, and magnitude of the fft(modal coordinates) recovered using L1 minimization after the principle component analysis for the case of randomly removing 90% of the frames. As indicated by the MAC results, the mode shapes are very similar to those recovered from the original video. In additional, the modal coordinates display the same frequency content and natural frequencies observed when analyzing the video in its original form. Figure 19 shows the mode shapes, modal coordinates, and the magnitude of the fft(modal coordinates) recovered using L1 minimization after the principle component analysis for the case of randomly removing 95% of the frames. As indicated by the MAC results, the 1st and 2nd mode shapes look similar to those recovered from the original video. The 3rd mode shape is beginning to become distorted as evidenced by the lower MAC value of only 0.92. In this case the modal coordinates’ peak frequencies correspond with the natural frequencies observed when analyzing the original video. However in the case of the second mode, the modal coordinate is beginning to take on frequency content that is not found in the modal coordinates recovered from the original video. Regardless, in the case of 90% of the frames randomly removed, the modal identification works quite well. This result suggests that when making video measurements in a noisy field environment. Recall the mode shape and MAC results shown in Figure 11 and Figure 12. In these results it is shown that the inclusion of even a very small number of corrupt frames in the analysis can result in a significant degradation in the performance of the structural system identification. In contrast, when L1 minimization is used to recover the principle components of the pixel times series a large percentage of the frames can be removed and the principle components will still be faithfully recovered. Therefore, from the perspective of the accuracy of the mode shape estimates and corresponding MAC values, it is often conservative to err on the side of removing potentially corrupt frames because so many frames can potentially be removed and a reasonable estimate of the modal parameters can still be obtained. In contrast, based on the mode shape and MAC results shown in Figure 11 and Figure 12, if even a small number of corrupt frames are allowed to be included in the analysis the result can be a complete failure of the structural system identification. This result should be considered when deciding which frames to use when field-deploying networks of video-based sensor nodes to monitor critical infrastructure. 

## 8. Recovering Mode Shapes from Sparse Samples of Frames without a Measurement Matrix or L1 Minimization

**Figure 20:**
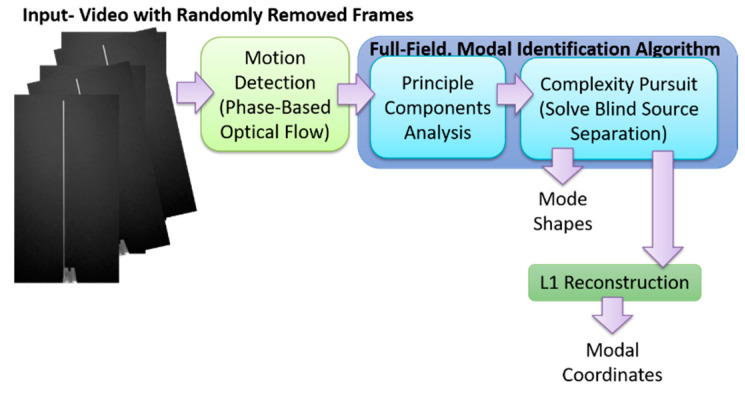
Algorithm for recovering mode shapes from video with frames randomly removed without the **need** for L1 regularization. L1 regularization is only used to recover the modal coordinates.

**Figure 21:**
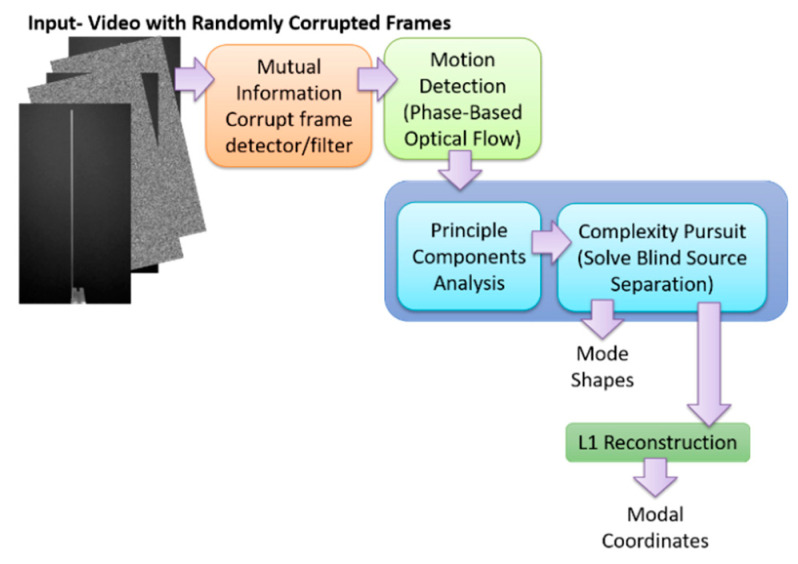
Algorithm for recovering mode shapes from video with randomly corrupted frames without using L1 regularization. L1 regularization is only used to recover the modal coordinates.

Now a technique is demonstrated for performing modal identification on video with frames randomly removed, in such a way that the mode shapes can be recovered without any need for knowledge of how the frames are randomly removed (e.g., measurement matrix), nor with any need for L1 minimization. In this approach knowledge of the measurement matrix is needed if a proper estimate of the modal coordinates is desired. Figure 20 and Figure 21 illustrate the procedure for performing full-field, high-resolution mode shape recovery from randomly sampled video. This is accomplished without using the measurement matrix or the L1 minimization process described in Section 3. The procedure is outlined for the case of randomly sampled frames, as well as for the case of video frames being randomly corrupted. These figures also show how to recover the modal coordinates using the measurement matrix as well as L1 regularization. The procedure for the two cases is identical except that in the case of the randomly corrupted frames the video is first run through the mutual information-based corrupt frame detection/filtering algorithm. In both cases, the remaining frames are then sent to the motion detection algorithm. In this work we only consider pixel time series associated with pixels that display motion in order to reduce the amount of data that must be processed. A list of the “active” pixel locations is maintained and used to reconstruct the total mode shape in pixel coordinates at the end of the analysis. The resulting motion time series at each pixel are then subjected to principle components analysis and a solver for the blind source separation problem (e.g., complexity pursuit). The mode shapes are then directly extracted from the columns of the mixing matrix and up-projected to the pixel space using the principle components matrix. The list of pixel locations associated with the “active” pixels is used to sort the mode shape components into their appropriate locations in the pixel space. All remaining “inactive” pixel locations are set to 0. In order to get the modal coordinates with the full length of 600 points to match the number of frames in the video, L1 minimization is used with the measurement matrix to reconstruct the full-length time series using the Fourier basis. 

**Figure 22:**
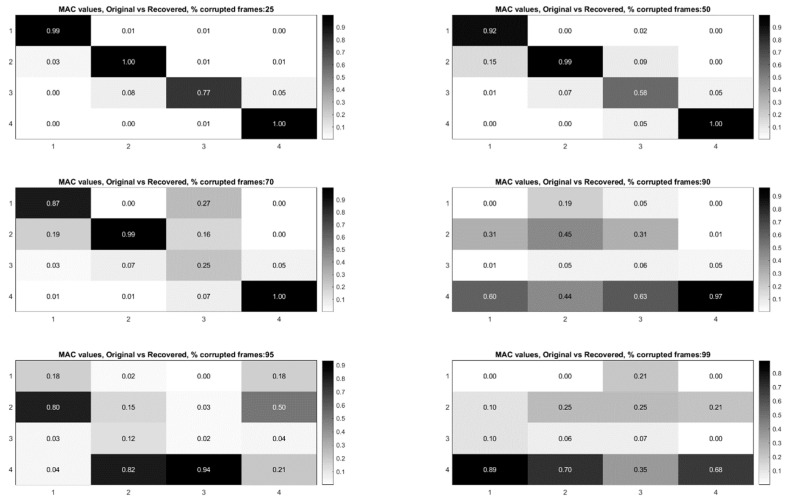
Modal assurance criterion values comparing the mode shapes recovered from the original video with the mode shapes recovered from the randomly sampled video without the use of L1 minimization.

**Figure 23:**
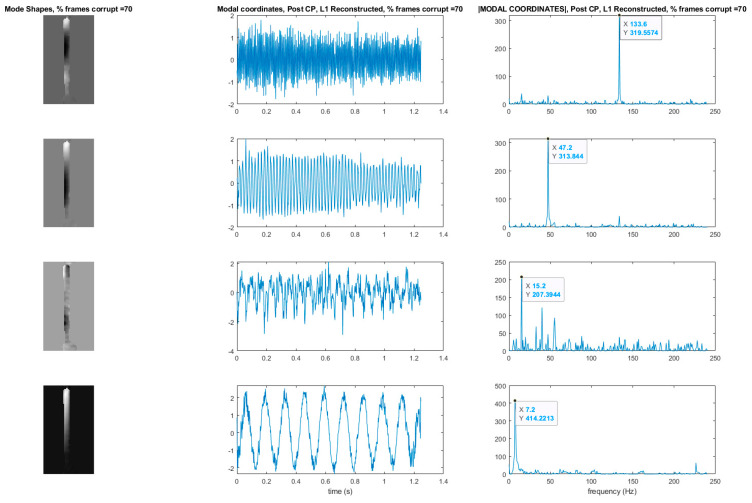
Mode shapes recovered from randomly sampled video without the of L1 minimization. Modal coordinates are recovered using L1 minimization on the output of the complexity pursuit algorithm. *Y*-axis of the modal coordinate and the magnitude of the fft(modal coordinate) plots are arbitrarily scaled displacement.

Figure 22 shows the modal assurance criterion values associated with the outlined recovery procedure for the cases of 25%, 50%, 70%, 90%, 95%, and 99% of the frames in the video randomly removed. In general the estimates of the modal coordinates recovered using L1 minimization after the blind source separation step are not of as high quality as when blind source separation is performed after the PCA step for the same percentage of frames removed. However, good mode shape identification can be achieved without any L1 reconstruction and no knowledge of the measurement matrix when as many as 70% of the frames are randomly removed (Figure 23). It is also clear that if L1 minimization is applied to the modal coordinates after the complexity pursuit step, that the recovered modal coordinates display the same frequency content and natural frequencies as is found in the analysis of the original video. The high-resolution, full-field algorithm can in some sense perform compressive sensing recovery simply as a result of the properties of linear structural dynamics and the form of the blind source separation problem.

It is worth noting that the modal identification results obtained when L1 minimization is not used are not as high of quality as those obtained when L1 minimization is used after the PCA step. However, these results are still noteworthy because they do not require any knowledge of how the frames are randomly sampled (i.e., measurement matric information is not needed), and these results demonstrate that L1 minimization is not needed to implement compressive sampling on video when all that is needed is the mode shapes of the vibrating structure. These results could possibly lead to new insights for compressive sensing hardware for structural dynamics as well as new possibilities for privacy-preserving structural health monitoring. 

## 9. Temporally Shuffling Frame Order

**Figure 24:**
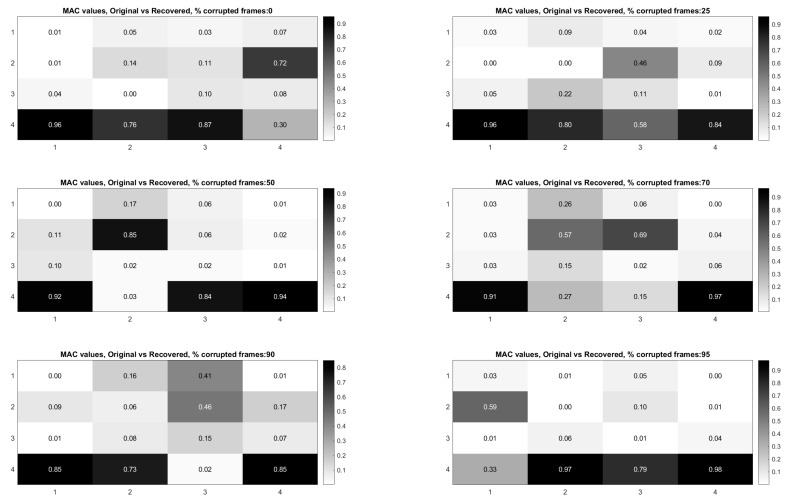
Modal assurance criterion values for the vibrating cantilever beam videos with randomly shuffled frame order and frames randomly removed.

**Figure 25:**
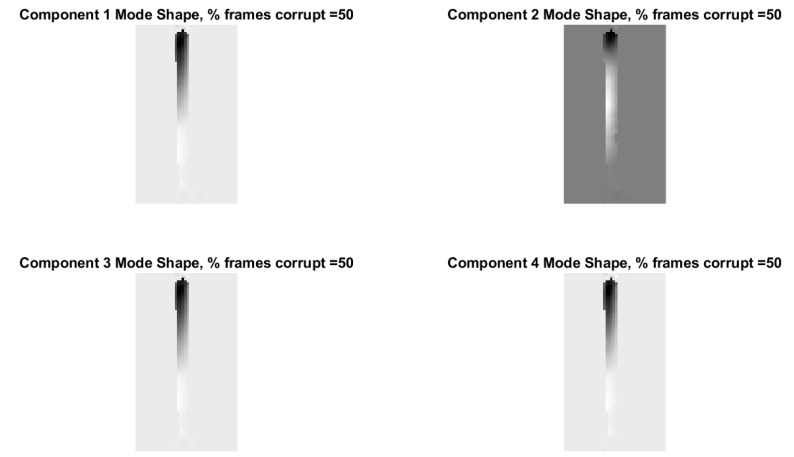
Mode shapes recovered when 50% of the frames and randomly removed and the frames are randomly shuffled in temporal order.

The previous section exploited the fact that the high-resolution mode shape information is a consequence of the mixing relationship between the pixel time series to show that mode shape information can be recovered from randomly sampled frames without any need for knowledge of the mixing matrix or the use of an L1 regularization algorithm to recover the mode shapes. The mixing relationship between the time series is preserved regardless of whether or not some of the frames are missing. An astute observer will also notice that the mixing relationship in the classical incarnation of the blind source separation problem is also invariant with respect to time. Theory would suggest that the temporal order of the frames could be completely shuffled and frames could be randomly removed, and it would still be possible to recover vibration mode shapes only using solvers for the blind source separation problem. To check the viability of this hypothesis the order of the frames in the video of the vibrating cantilever beam were randomly shuffled and then frames were randomly removed. The videos were then subjected to the full-field, high resolution decomposition algorithm for modal identification. The modal assurance criterion values for the mode shapes recovered from the randomly shuffled and randomly sampled videos are found in Figure 24. For the most part only the first mode is recovered, however, in some cases a reasonable estimate of the second mode is recovered as well. This is best illustrated in the case where 50% of the frames are removed. The mode shapes associated with 50% frame removal and random shuffling of frame order are shown in Figure 25. In this case the second component recovered by the blind source separation solver does resemble the second mode shape. The point of this analysis is not to suggest that structural dynamists should randomly shuffle the order of their frames when performing analysis. Clearly the performance of the modal identification suffers greatly when the temporal order is removed. Instead the point is to illustrate the surprising result that 50% of the frames in a video could be removed and then the order of the frames could be randomly shuffled and it was still possible to recover reasonable estimates of the first two mode shapes. This result suggests that variations of full-field, high-resolution decomposition algorithms for modal identification could be developed to be robust for situations where multiple multi-media sensor nodes are capturing measurements of a structure from different perspectives, but the temporal synchronization between the sensor nodes is poor. Furthermore, this random shuffling of frame order suggests ways that structural dynamics can be combined with cryptography as will be discussed in the next section. 

## 10. Results Spatially Shuffling the Pixel Order

A careful examination of the blind-source separation approach for full-field, high resolution modal identification reveals that it is also possible to randomly shuffle the order of the pixels (after the motion detection step) and still obtain the same mode shapes and modal coordinates assuming a list of the pixel shuffling indices is maintained to rearrange the randomly shuffled mode shape back into the original pixel space at the end of the analysis. This is because no spatial information is needed for obtaining the mode shapes. The mode shapes are found using only the mixture matrix that relates the modal coordinate time series to the projection of the measured time series onto the dimensionality reduction matrix associated with the principle components analysis step. In fact unlike the case of randomly shuffling the frames, no degradation in performance is observed when randomly shuffling the pixel order. It is currently hypothesized that the degradation in performance associated with the random shuffling of the frames is an artifact of the numerical technique (complexity pursuit), used to find the solution to the blind source separation problem. It is possible that solutions to blind source separation exist or can be developed that mitigate the loss in performance, but finding such a technique can be considered in future work.

**Figure 26:**
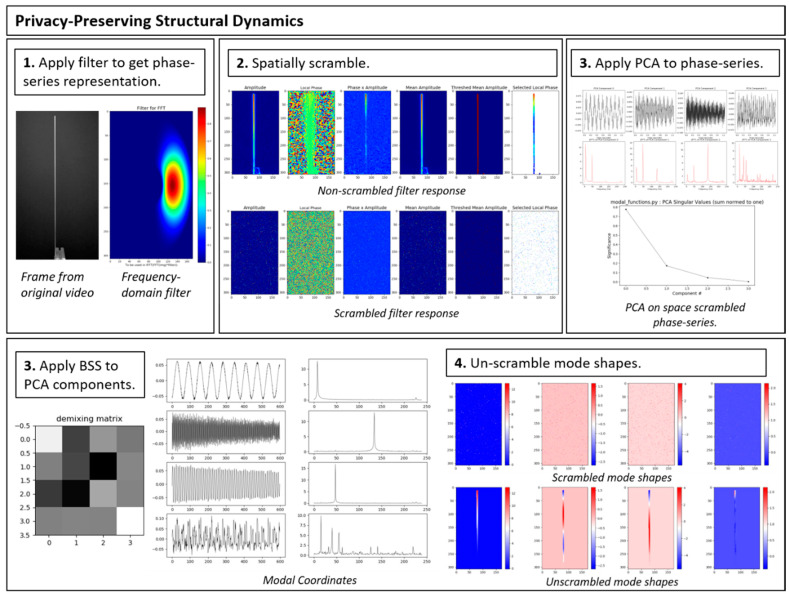
Demonstration of privacy-preserving structural dynamics identification.

The fact that randomly shuffling the spatial pixel order does not impact the ability to extract mode shapes and modal coordinates opens up some interesting possibilities for enabling privacy-preserving structural health monitoring and process monitoring. The problem of taking the randomly shuffling pixels and trying to rearrange them back into the original images is an example of the computational problem known as the “jigsaw puzzle problem.” It is actually a very extreme form of the jigsaw puzzle problem because the picture is broken down all the way into individual pixels. It has been shown that the jigsaw puzzle problem is NP-complete [74]. This suggests that randomly shuffling pixels may be an effective encryption technique for structural dynamics applications. Future work should look at the possibilities associated with combining data shuffling (both spatial and temporal) with for the purpose of enabling homomorphic computation of modal identification. If a shuffling technique can be made that allows accurate modal information to be computed from shuffled data it would enable a form a privacy-preserving computing for structural dynamics. Shuffled data that is effectively encrypted could be sent from a multi-media sensor node to a cloud service for more intensive computations. The cloud service could perform its computations with no ability to know any spatial information regarding the structure it is analyzing. It could then send the analysis results back to the multi-media sensor node which can use its secret knowledge of the shuffling order to get back the mode shape of interest in the original pixel coordinates. Figure 26 provides a demonstration of how privacy-preserving structural dynamics identification can be carried out using the procedure described here on the vertical cantilever beam. This demonstration was carried out with the help of the utility files for complex steerable pyramids provided by Zhou [57]. The ability to securely transmit structural dynamics information to cloud services for intensive computation could be particularly important for applications such as analyzing medical data as well as in-process monitoring of manufacturing processes. The development of such a scheme would be an interesting interplay between cryptography, structural dynamics and compressive sensing. In fact, shuffling pixel order can be implemented by multiplying a vertical vector of pixel intensity values by a random matrix consisting of 0 and 1 entries with only a single value of 1 in each row. This type of random matrix is quite similar to a random Dirac basis matrix that can be used in compressive sampling, thus suggesting a relationship between the security properties associated with compressive sampling coefficients and the jigsaw puzzle problem. It is worth noting that compressed sensing measurements themselves have been shown to possess computational security properties [75]. This privacy-preserving homomorphic encryption scheme would be particularly useful as 5G networks become increasingly available. 

## 11. Conclusions

This work presents techniques for combining random sampling techniques with full-field high-resolution modal identification algorithms for purposes of data compression, denoising and privacy-preservation. A normalized mutual information technique is presented for detecting the presence of corrupted frames so they can be removed from consideration during modal identification. The paper presents a technique for recovering mode shapes from randomly sampled frames from video of vibrating structures that involve the use of L1 minimization algorithms as are typically used in compressive sampling as well as a technique that does not require L1 minimization. The paper also presents two strategies for recovering modal coordinates from randomly sampled frames from a video of a vibrating structure using L1 minimization algorithms. Interestingly, the results of this work suggest that under noisy field conditions it is generally conservative from the perspective of the MAC values of the mode shape estimates to remove frames from video that might be corrupted rather than to possibly allow them to remain and corrupt the modal identification analysis. Finally, the paper demonstrates how mode shapes can be recovered from randomly sampled, and shuffled frames captured from a video of a vibrating structure. The paper presents the reader with information on the tradeoffs in performance that are associated with the different approaches to using compressive sampling for video dynamics techniques in order to better select a specific approach for a given structural health monitoring application. The results of this work point to future applications such as privacy-preserving structural health monitoring, reducing phototoxicity/photobleaching effects during imaging of live cells for biological applications, and data compression for long-term monitoring of critical infrastructure such as wind turbines and bridges. This work also provides a path forward for research into learning high-dimensional models from small numbers of training examples.

An interesting feature of the structural dynamics system identification technique presented here is that the unsupervised learning takes place directly over data that is represented in the randomly subsampled Dirac basis. It is shown that system identification can take place over far fewer samples than would be captured using conventional sampling schemes. This observation is important because contemporary deep-learning techniques tend to require large amounts of training data to achieve high levels of performance. Compressive sampling techniques combined with unsupervised learning may provide a path toward more general learning schemes that do not require large amounts of training data. 

The techniques described in this work are demonstrated to be able to recover mode shapes and modal coordinates from experimental video of vibrating structures when 70% to 90% of the frames from a video captured in the conventional manner are removed. The ability to remove such large percentages of frames and still be able to recover mode shapes makes blind source separation–based modal analysis techniques particularly attractive for deployment in video-based, low-power, wireless sensor networks that utilize emerging 5G network communication infrastructure for structural health monitoring applications. These techniques help work toward enabling video-based wireless sensor networks for structural health monitoring applications in real-world environments because the compression offered by the techniques in this paper greatly reduce the amount of data that potentially needs to be transmitted to central servers or cloud computing resources that aggregate data from across the sensor network. The results are lower bandwidth requirements, and less energy consumption associated with transmitting the data. Furthermore, the demonstrations of randomly shuffling the order of the pixels and the frames provide the ability to perform structural identification in a privacy-preserving mode which enhances the security of the over-all structural health monitoring system which is an important issue when monitoring critical infrastructure. Security is a known concern with emerging high-performance 5G networks, and these concerns must be addressed for critical infrastructure monitoring applications. This work also point to a path forward for learning high-dimensional models from a small number of training examples.
